# Hypertrophic Scar Formation Following Burns and Trauma: New Approaches to Treatment

**DOI:** 10.1371/journal.pmed.0040234

**Published:** 2007-09-04

**Authors:** Shahram Aarabi, Michael T Longaker, Geoffrey C Gurtner

## Abstract

The authors examine the process of hypertrophic scar formation, the results of current treatments, and areas of research likely to lead to significant advances in the field.

Hypertrophic scar formation is a major clinical problem in the developing and industrialized injuries, and surgical procedures can give rise to exuberant scarring that resaults in permanent functional loss and the stigma of disfigurement. Figure 1 illustrates the scope of the problem. Annually, over 1 million people require treatment for burns in the United States [1], 2 million are injured in motor vehicle accidents [2], and over 34 million related surgical procedures are performed [3]. Although the incidence of hypertrophic scarring following these types of injuries is not known, it is a common outcome that creates a problem of enormous magnitude. Treatment of these cases is estimated to cost at least $4 billion per annum in the US alone [4]. The incidence of burns and traumatic injuries is even greater in the developing world [5]. This review will examine the process of hypertrophic scar formation, the results of current treatments, and areas likely to lead to significant advances in the field.

**Figure 1:**
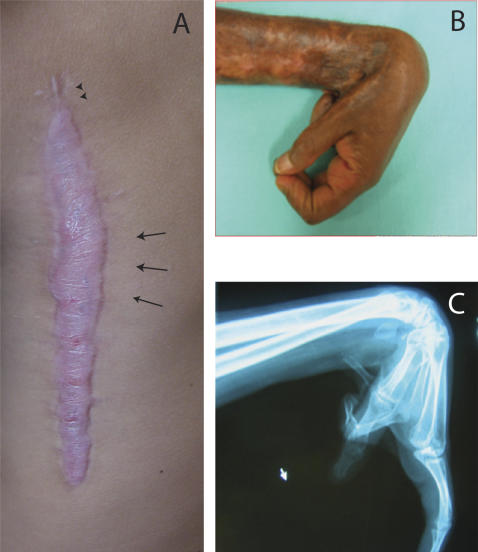
Complications of Hypertrophic Scarring (A) Hypertrophic scars begin as small cutaneous fibrotic regions (arrowheads), which develop into gross scars (arrows) over time. Scarring phenotypes vary widely between different parts of the body for reasons that are at present unclear. (B) Following burn injury, a patient shows severe joint contracture. (C) Radiograph of the same patient shows erosion of the bone secondary to disuse and contracture. After years of treatment and physical therapy, this patient will only regain minimal hand function.

## Evolution of Patient Care

Advances over the past 60 years have allowed us to extend the lives of patients whose injuries would previously have been invariably fatal. Fire disasters such as those at the Rialto concert hall (1930) [6] and the Cocoanut Grove nightclub (1942) [7] led to the development of new treatments, such as fluid resuscitation, to prevent death in the early stages following burn injury. World War II led to the development of critical care medicine [8], further improving the ability to keep those with traumatic injuries alive until surgical management of their wounds was possible. Antibiotics and aggressive surgical debridement have also contributed to the survival of the great majority of burn and trauma patients. However, despite advances in life-saving technology, progress to prevent the late functional and aesthetic sequelae of hypertrophic scar formation has been slow [9].

Efforts to limit scar formation in burn and trauma patients have relied largely on immediate skin replacement [10] with human split-thickness allografts or dermal analogs such as Integra. Although these measures provide excellent barriers against infection and mechanical trauma, the long-term improvement in appearance has been modest [11,12]. After healing has occurred, massage, pressure therapies, steroids, and silicone dressings are frequently used to manage the massive scar burden in these patients [13]. Many of these treatments predate modern medicine and their benefits remain unclear [11]. As stated in a major review on burns and scarring, even with state-of-the-art care, “hypertrophic scarring remains a terrible clinical problem” [11].

One barometer of the futility of these attempts at scar modulation is the interest in total facial transplantation. This procedure has been suggested as a measure of last resort for patients with severe facial disfigurement due to scar formation [14,15]. However, facial transplantation has sparked controversy due to the severe antigenicity of allograft skin used and side effects of the antirejection medications required. It is a testament to the intractability of this problem that such desperate measures are currently being considered. When full facial transplantation is eventually performed, it is likely that the recipient will be a patient with facial burns and the resulting functional deficits and stigmata of hypertrophic scar formation.

**Five Key Papers in the Field**

**Aarabi et al., 2007** [74] Demonstrates that mechanical stress is necessary to replicate hypertrophic scar formation in the first murine model of the disease.

**Ting et al., 2005** [58] Demonstrates that the mechanisms regulating skin repair are evolutionally conserved over millions of years.

**Shah et al., 1992** [39] Demonstrates that inhibiting inflammatory mediators such as TGF-ß can reduce scar formation in vivo.

**Burrington, 1971** [26] A seminal paper in the study of scar formation versus regeneration where it was first demonstrated that fetal wounds heal without scar in utero.

**Majno et al., 1971** [57] Illustrates that fibroblasts take on contractile properties during wound healing, suggesting that cutaneous healing may occur in a mechanically unique environment.

## Pathophysiology

Clinical experience suggests that hypertrophic scarring is an aberrant form of the normal processes of wound healing [16]. However, the etiology of the overexuberant fibrosis is unknown. Hypertrophic scarring should be distinguished from keloid formation, the other major form of excessive scarring seen in humans. There is stronger evidence for genetic predisposition in keloid formation than in hypertrophic scarring, although both occur more frequently in certain groups (e.g., people of African and Asian descent). Keloids are characterized by overgrowth of fibrosis beyond the boundaries of the original injury, while hypertrophic scars do not extend beyond the original wound margins. Keloids and hypertrophic scars can also be differentiated by established histopathological criteria, which include differences in collagen density and orientation, vascularity, and other factors [17,18].

The pathophysiology of hypertrophic scar formation involves a constitutively active proliferative phase of wound healing [16]. Scar tissue has a unique structural makeup that is highly vascular, with inflammatory cells and fibroblasts contributing to an abundant and disorganized matrix structure [16]. The net result is that the original skin defect is replaced by a nonfunctional mass of tissue. Beyond these observations, investigations into the pathophysiology of the disease have been limited by the absence of a practical animal model and have relied upon the use of human pathological specimens [19–21]. These studies are problematic in that such specimens represent the terminal stages of the scarring process and may not contain the initiating factors that originally led to the development of the disease. The few animal models that have been used include the rabbit ear [22] and the red Duroc pig [23]. While they have given us some insight into the genetics and pathogenesis of cutaneous fibrosis [24,25], it is unclear how closely the process of hypertrophic scarring in these models resembles that seen in humans. Specifically, it is unknown whether the same factors that initiate hypertrophic scarring in these species are involved in human disease. Further, studies using these species have been limited by a paucity of molecular reagents available for rabbits and pigs. For the aforementioned reasons, these observational studies have not resulted in notable therapeutic advances.

Fetal wound healing has been proposed as a vehicle to study skin regeneration. Early fetal wound healing is characterized by the complete absence of scar formation [26]. The developing fetus transitions to a scarring phenotype during the third trimester of gestation [27]. During the scarless phase of development, both low fibroblast activity and a decreased inflammatory response to injury are observed [27]. Experiments have shown that local factors in wounded skin, rather than systemic or maternal factors, are responsible for this transition from scarless to scarred healing [28–31]. However, it is unclear which local factors in the wound initiate scar formation and which are secondary to the scarring process. Thus it has been difficult to separate cause from effect using the fetal wound model.

In both adult and fetal healing, the local wound environment interacts with the cellular components of wound healing and vice versa. The local wound environment consists of noncellular influences such as matrix components, oxygen tension, and mechanical forces. The interplay between cellular (“seed”) and noncellular (“soil”) components is complex, with constant feedback between the two during the healing process (Figure 2). Many therapies for hypertrophic scar formation may underestimate this complexity by focusing on a single component of this relationship. Tables 1 and 2 provide a review of the multitude of established and experimental therapies and their proposed mechanisms of action. To date, none of these approaches have achieved wide clinical adoption [11].

**Figure 2:**
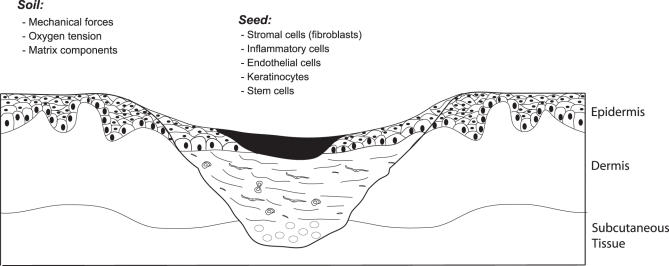
Seed versus Soil Cellular and noncellular factors both play a role during the process of scar formation. Local environmental factors such as mechanical forces, extracellular matrix structure and orientation, and oxygen tension act as cellular signals. These signals influence the migration, adhesion, extravasation, and proliferation of varied cell types. These cells respond and in turn alter the physicochemical environment in which they reside. Keratinocytes migrate and multiply, changing the mechanical structure along the wound margin. Fibroblasts increase matrix production and initiate remodeling. Endothelial cells take part in neovascularization and regulate the blood flow and oxygen tension in the wound. As these cells alter their environment, complex feedback mechanisms move the wound healing process through its normal inflammatory, proliferative, and remodeling phases. Aberrant wound healing occurs when environmental or cellular factors are altered. Increased mechanical tension or oxygen dysregulation, for example, can lead to a constitutively active proliferative phase, increased matrix deposition, and hypertrophic scar formation.

**Table 1:**
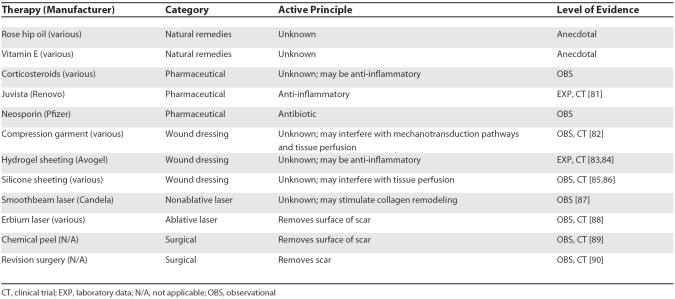
Selection of Currently Available Therapeutics for the Treatment of Hypertrophic Scarring

**Table 2:**
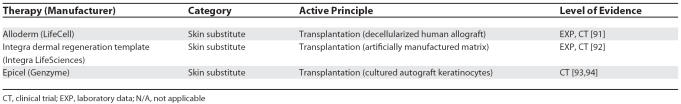
Selection of Currently Available Therapeutics for the Prevention of Hypertrophic Scarring

It is unclear whether changes in the seed or soil are responsible for the phenomenon of hypertrophic scar formation. When compared to fetal wound healing, adult wound healing is a response to injury that sacrifices the regeneration of original tissue for a rapid matrix plug, or scar, that protects the organism from infection and trauma [16]. This response is evolutionarily conserved and allows the adult organism to survive despite the harsh extrauterine environment. However, the possibility exists that regenerative capacity can be restored in adults, and that wound healing could proceed with a recapitulation of the original skin architecture rather than with the patching characteristic of scar formation. In the next section we will consider existing and proposed therapies for hypertrophic scar formation using this framework.

## Therapeutic Approaches: Targeting Inflammatory Mediators

The inflammatory response is a normal component of the wound healing process, serving both as an immunological barrier from infection and as a stimulus for fibrosis to close the site of injury. Observations from human pathological specimens and from healing fetal wounds suggest that a robust inflammatory response may underlie the excessive fibrosis seen in hypertrophic scar formation [16,18]. Mast cells, macrophages, and lymphocytes have all been implicated in this process [16,18]. For example, mast cells have been shown to directly regulate stromal cell activity in vitro [32] as well as to be strongly associated with the induction of fibrosis in vivo [33]. Mechanical activity, age-specific changes, and delayed epithelialization have all been implicated as inciting factors for this intense inflammatory response.

While the phenomenology of the myriad cytokines involved in wound healing is vast, the discussion of some key regulators of the scarring process is unavoidable. Following cutaneous injury, endothelial damage and platelet aggregation occur resulting in the secretion of cytokines including the transforming growth factor (TGF)-ß family, platelet-derived growth factors (PDGF), and epidermal growth factors (EGF) [11,16]. These cytokines stimulate fibroblast proliferation and matrix secretion, and induce leukocyte recruitment. Leukocytes, in turn, reinforce fibroblast activity, fight infection, and increase vascular permeability and ingrowth. They do this acting through the TGF-ß family, fibroblast growth factors (FGF), vascular endothelial growth factors (VEGF), and other factors [11,16]. Prostaglandins [34] and SMAD activation [35] also increase inflammatory cell proliferation and impair matrix breakdown [36]. Increased levels of TGF-ß1 and ß2 as well as decreased levels of TGF-ß3 have been associated with hypertrophic scarring through inflammatory cell stimulation, fibroblast proliferation, adhesion, matrix production, and contraction [37,38]. Consistent with these observations, anti-inflammatory agents (cytokine inhibitors, corticosteroids, interferon a and ß, and methotrexate) have been used with some success to reduce scar formation [11,39]. Novel antifibrotic agents are also in development to target specific mediators of the scarring process [40,41].

Increased vascular density, extensive microvascular obstruction, and malformed vessels [25,42] have also been observed in hypertrophic scars. These structural changes may account for the persistent high inflammatory cell density observed in hypertrophic scars. Conversely, persistent inflammation could itself contribute to increased vascularity through positive feedback loops. Although the presence of a robust inflammatory response during scar formation has been described, many questions remain unanswered. Specifically, what distinguishes physiological or “normal” inflammation from the pathological inflammation that occurs during hypertrophic scar formation? What signals act to initiate or stop this excessive inflammatory process in scar formation? Until these issues are clarified it will be difficult to ascertain what causal roles inflammatory pathways have in initiating hypertrophic scar formation.

## Therapeutic Approaches: Targeting Epithelial–Mesenchymal Interactions

Epithelial cells have important roles in normal skin physiology, which include acting as stem cell niches and participating in complex signaling pathways to regulate mesenchymal cell function. The net results of these functions are the constant renewal of skin layers and the regulation of matrix deposition and remodeling. Cell-based skin substitutes take advantage of the regenerative nature of skin and are clinically used to cover wounds, but their utility in subsequent scar formation remains unknown. Epidermal stem cells are thought to act in concert with mesenchymal cells in the dermal papillae, functioning to recruit new cells to sites of skin regeneration [43,44]. However, large traumatic skin defects (such as those following burn injuries) destroy the resident epidermal stem cell population and cannot be spontaneously regenerated.

Efforts to isolate and purify epidermal stem cells in order to prepare them for ex vivo expansion and subsequent transplantation require the identification of surface markers specific to these cells [45,46]. Elucidation of these markers has been challenging, but work is progressing [43] and will hopefully soon yield methods to easily obtain pure populations of cells with high proliferative potential.

In addition to their regenerative function, epithelial cells act to modulate mesenchymal cell proliferation and activity in normal skin and during wound healing and scar formation [47]. In healing wounds, epithelial cells promote fibrosis and scarring through multiple pathways including SMAD, phosphoinositide-3 kinase (PI3K), TGF-ß, and connective tissue growth factor (CTGF) [48–51]. Epithelial cells stimulate fibroblasts during hypertrophic scar formation and fibroblasts themselves undergo intrinsic changes during the process of scarring [52–54]. Subsequently, fibroblasts remain in an activated state, participating in cytokine autocrine loops that maintain fibrosis [52–56]. Hypertrophic scar fibroblasts also have fundamentally altered profiles of cellular apoptosis, matrix production, and matrix degradation [52–56]. It is unclear whether these altered, profibrotic properties are due to genetic predisposition or secondary to unique conditions present in the wound environment.

## Therapeutic Approaches: Targeting the Physical Environment

Following injury, the wound is a complex and mechanically unique environment [57,58] with multiple levels of interaction between cells and the surrounding milieu. Fibroblasts and keratinocytes respond to the density and orientation of collagen and other matrix components [59–61]. As a result, cells near the wound margin proliferate while those further away from the edge of the wound are less active [62,63]. At the same time, these cells are actively producing and remodeling the surrounding matrix. It is this delicate balance that is responsible for a rapid and healthy response to injury and, when disturbed, leads to aberrant wound healing.

Many cells are known to be mechanoresponsive [64,65]. It has recently become clear that cells in the skin are also able to respond to their mechanical environment [66–68]. Specifically, cell surface molecules such as the integrin family are activated by mechanical forces, leading to increased fibroblast survival as well as to the remodeling of deposited collagen and fibrin [66,69]. While the intracellular signaling involved in this process is complex and incompletely understood, transcriptional regulators such as AKT and focal adhesion kinase (FAK) have been found to be essential elements [66,69,70]. Keratinocyte proliferation and migration are similarly regulated by mechanical stress [67,71]. Following tissue injury, mechanotransduction may serve a biological function to signal the presence of a tissue defect. Cells experience the highest levels of mechanical stress on the edge of a monolayer [72] and, in the same way, the wound margin experiences high levels of mechanical stress [73]. These stresses may have evolved to stimulate components of wound healing and initiate repair. Differences in exogenous forces may act to change cellular activation in the wound healing milieu and, when overactivated, lead to hypertrophic scar formation [74]. Clinically, we see that these expectations hold true. Skin subjected to high levels of stress (secondary to trauma or joint movement) usually demonstrates robust hypertrophic scar formation [27,75].

Oxygen tension is another component of the physical environment that may be important for scar formation. Changes in levels of the transcription factor hypoxia-inducible factor (HIF)-1a during fetal skin development are thought to be partly responsible for the transition from scarless to scarred healing [76,77]. Varying levels of HIF-1a in turn result in changes in a number of downstream proteins including TGF-ß3 and VEGF [76,78]. Changes in hypoxia signaling pathways contribute to the maturation of fetal skin and the development of a scarring phenotype following wounding [77,78]. Changes in oxygen tension and increases in reactive oxygen species have also been shown to mediate early scar formation in tissues such as the lung and heart [79,80]. However, the observation that scars are normally highly vascular is at odds with the theory that hypoxia increases scar formation, and further work is needed to definitely establish this relationship. What is clear is that the wound environment is a powerful modulator of scar formation and could potentially be manipulated for therapeutic effect.

## Conclusion

The complex interplay between cell influx into the wound bed, environmental factors in the surrounding skin, and various cytokine mediators makes the task of manipulating the wound environment to promote regeneration appear daunting. Presently, most therapies consist of a single cell type or cytokine being added to the healing wound in the hopes that this will result in perfect healing. As we have described, monotherapy is unlikely to be effective. However, it is equally improbable that the entire web of factors that promote tissue regeneration can be incorporated into a single therapeutic strategy. It is likely that the development of more effective therapeutics will require an incorporation of known environmental factors along with cellular components to promote healing. A comprehensive strategy taking into account both the cellular (seed) and environmental (soil) contributions to hypertrophic scar formation will have the highest likelihood of therapeutic success against this currently incurable condition.

## Abbreviations

TGF: transforming growth factor

## References

[pmed-0040234-b001] Brigham PA, McLoughlin E (1996). Burn incidence and medical care use in the United States: Estimates, trends, and data sources. J Burn Care Rehabil.

[pmed-0040234-b002] National Highway Traffic Safety Administration (2004). Traffic safety facts 2004. http://www-nrd.nhtsa.dot.gov/pdf/nrd-30/NCSA/TSFAnn/TSF2004.pdf.

[pmed-0040234-b003] Merrill CT, Elixhauser A (2003). Healthcare Cost and Utilization Project: Procedures in U.S. hospitals, 2003. http://www.ahrq.gov/data/hcup/factbk7/.

[pmed-0040234-b004] MedMarket Diligence (2005). Worldwide wound management, 2005–2014: Established and emerging products, technologies and markets in the U.S., Europe, Japan and rest of world.

[pmed-0040234-b005] WHO (2002). Global Burden of Disease Project. http://www.who.int/healthinfo/bodproject/en/index.html.

[pmed-0040234-b006] Underhill F (1930). The significance of anhydremia in extensive superficial burns. JAMA.

[pmed-0040234-b007] Saffle JR (1993). The 1942 fire at Boston's Cocoanut Grove nightclub. Am J Surg.

[pmed-0040234-b008] Safar P (1996). On the history of modern resuscitation. Crit Care Med.

[pmed-0040234-b009] Sheridan RL (2003). Burn care: Results of technical and organizational progress. JAMA.

[pmed-0040234-b010] Atiyeh BS, Hayek SN, Gunn SW (2005). New technologies for burn wound closure and healing—Review of the literature. Burns.

[pmed-0040234-b011] Sheridan RL, Tompkins RG (2004). What's new in burns and metabolism. J Am Coll Surg.

[pmed-0040234-b012] van Zuijlen PP, Vloemans JF, van Trier AJ, Suijker MH, van Unen E (2001). Dermal substitution in acute burns and reconstructive surgery: A subjective and objective long-term followup. Plast Reconstr Surg.

[pmed-0040234-b013] Mustoe TA, Cooter RD, Gold MH, Hobbs FD, Ramelet AA (2002). International clinical recommendations on scar management. Plast Reconstr Surg.

[pmed-0040234-b014] Hettiaratchy S, Butler PE (2002). Face transplantation—Fantasy or the future?. Lancet.

[pmed-0040234-b015] McDowell N (2002). Surgeons struggle with ethical nightmare of face transplants. Nature.

[pmed-0040234-b016] Singer AJ, Clark RA (1999). Cutaneous wound healing. N Engl J Med.

[pmed-0040234-b017] Ehrlich HP, Desmouliere A, Diegelmann RF, Cohen IK, Compton CC (1994). Morphological and immunochemical differences between keloid and hypertrophic scar. Am J Pathol.

[pmed-0040234-b018] White CR, Barnhill RL, Crowson AN (2004). Textbook of dermatopathology.

[pmed-0040234-b019] Eddy RJ, Petro JA, Tomasek JJ (1988). Evidence for the nonmuscle nature of the “myofibroblast” of granulation tissue and hypertropic scar. An immunofluorescence study. Am J Pathol.

[pmed-0040234-b020] Lewis WH, Sun KK (1990). Hypertrophic scar: A genetic hypothesis. Burns.

[pmed-0040234-b021] Savage K, Swann DA (1985). A comparison of glycosaminoglycan synthesis by human fibroblasts from normal skin, normal scar, and hypertrophic scar. J Invest Dermatol.

[pmed-0040234-b022] Morris DE, Wu L, Zhao LL, Bolton L, Roth SI (1997). Acute and chronic animal models for excessive dermal scarring: Quantitative studies. Plast Reconstr Surg.

[pmed-0040234-b023] Zhu KQ, Engrav LH, Gibran NS, Cole JK, Matsumura H (2003). The female, red Duroc pig as an animal model of hypertrophic scarring and the potential role of the cones of skin. Burns.

[pmed-0040234-b024] Reid RR, Mogford JE, Butt R, deGiorgio-Miller A, Mustoe TA (2006). Inhibition of procollagen C-proteinase reduces scar hypertrophy in a rabbit model of cutaneous scarring. Wound Repair Regen.

[pmed-0040234-b025] Zhu KQ, Engrav LH, Armendariz R, Muangman P, Klein MB (2005). Changes in VEGF and nitric oxide after deep dermal injury in the female, red Duroc pig-further similarities between female, Duroc scar and human hypertrophic scar. Burns.

[pmed-0040234-b026] Burrington JD (1971). Wound healing in the fetal lamb. J Pediatr Surg.

[pmed-0040234-b027] Ihara S, Motobayashi Y, Nagao E, Kistler A (1990). Ontogenetic transition of wound healing pattern in rat skin occurring at the fetal stage. Development.

[pmed-0040234-b028] Armstrong JR, Ferguson MW (1995). Ontogeny of the skin and the transition from scar-free to scarring phenotype during wound healing in the pouch young of a marsupial, Monodelphis domestica. Dev Biol.

[pmed-0040234-b029] Bullard KM, Longaker MT, Lorenz HP (2003). Fetal wound healing: Current biology. World J Surg.

[pmed-0040234-b030] Longaker MT, Whitby DJ, Ferguson MW, Lorenz HP, Harrison MR (1994). Adult skin wounds in the fetal environment heal with scar formation. Ann Surg.

[pmed-0040234-b031] Lorenz HP, Longaker MT, Perkocha LA, Jennings RW, Harrison MR (1992). Scarless wound repair: A human fetal skin model. Development.

[pmed-0040234-b032] Moyer KE, Saggers GC, Ehrlich HP (2004). Mast cells promote fibroblast populated collagen lattice contraction through gap junction intercellular communication. Wound Repair Regen.

[pmed-0040234-b033] Hermes B, Welker P, Feldmann-Boddeker I, Kruger-Krasagakis S, Hartmann K (2001). Expression of mast cell growth modulating and chemotactic factors and their receptors in human cutaneous scars. J Invest Dermatol.

[pmed-0040234-b034] Wilgus TA, Bergdall VK, Tober KL, Hill KJ, Mitra S (2004). The impact of cyclooxygenase-2 mediated inflammation on scarless fetal wound healing. Am J Pathol.

[pmed-0040234-b035] Saika S, Ikeda K, Yamanaka O, Flanders KC, Okada Y (2006). Loss of tumor necrosis factor alpha potentiates transforming growth factor beta-mediated pathogenic tissue response during wound healing. Am J Pathol.

[pmed-0040234-b036] Gorvy DA, Herrick SE, Shah M, Ferguson MW (2005). Experimental manipulation of transforming growth factor-beta isoforms significantly affects adhesion formation in a murine surgical model. Am J Pathol.

[pmed-0040234-b037] Dabiri G, Campaner A, Morgan JR, Van De Water L (2006). A TGF-beta1-dependent autocrine loop regulates the structure of focal adhesions in hypertrophic scar fibroblasts. J Invest Dermatol.

[pmed-0040234-b038] Kopp J, Preis E, Said H, Hafemann B, Wickert L (2005). Abrogation of transforming growth factor-beta signaling by SMAD7 inhibits collagen gel contraction of human dermal fibroblasts. J Biol Chem.

[pmed-0040234-b039] Shah M, Foreman DM, Ferguson MW (1992). Control of scarring in adult wounds by neutralising antibody to transforming growth factor beta. Lancet.

[pmed-0040234-b040] Reid RR, Roy N, Mogford JE, Zimmerman H, Lee C (2007). Reduction of hypertrophic scar via retroviral delivery of a dominant negative TGF-beta receptor II. J Plast Reconstr Aesthet Surg.

[pmed-0040234-b041] Braddock M (2005). Euroconference on tissue repair and ulcer/wound healing: Molecular mechanisms, therapeutic targets and future directions. Expert Opin Investig Drugs.

[pmed-0040234-b042] Clark JA, Leung KS, Cheng JC, Leung PC (1996). The hypertrophic scar and microcirculation properties. Burns.

[pmed-0040234-b043] Alonso L, Fuchs E (2003). Stem cells of the skin epithelium. Proc Natl Acad Sci U S A.

[pmed-0040234-b044] Paladini RD, Takahashi K, Bravo NS, Coulombe PA (1996). Onset of re-epithelialization after skin injury correlates with a reorganization of keratin filaments in wound edge keratinocytes: Defining a potential role for keratin 16. J Cell Biol.

[pmed-0040234-b045] Cotsarelis G, Sun TT, Lavker RM (1990). Label-retaining cells reside in the bulge area of pilosebaceous unit: Implications for follicular stem cells, hair cycle, and skin carcinogenesis. Cell.

[pmed-0040234-b046] Rochat A, Kobayashi K, Barrandon Y (1994). Location of stem cells of human hair follicles by clonal analysis. Cell.

[pmed-0040234-b047] Niessen FB, Andriessen MP, Schalkwijk J, Visser L, Timens W (2001). Keratinocyte-derived growth factors play a role in the formation of hypertrophic scars. J Pathol.

[pmed-0040234-b048] Verrecchia F, Mauviel A (2002). Transforming growth factor-beta signaling through the Smad pathway: Role in extracellular matrix gene expression and regulation. J Invest Dermatol.

[pmed-0040234-b049] Colwell AS, Phan TT, Kong W, Longaker MT, Lorenz PH (2005). Hypertrophic scar fibroblasts have increased connective tissue growth factor expression after transforming growth factor-beta stimulation. Plast Reconstr Surg.

[pmed-0040234-b050] Daniels JT, Schultz GS, Blalock TD, Garrett Q, Grotendorst GR (2003). Mediation of transforming growth factor-beta(1)-stimulated matrix contraction by fibroblasts: A role for connective tissue growth factor in contractile scarring. Am J Pathol.

[pmed-0040234-b051] Khoo YT, Ong CT, Mukhopadhyay A, Han HC, Do DV (2006). Upregulation of secretory connective tissue growth factor (CTGF) in keratinocyte-fibroblast coculture contributes to keloid pathogenesis. J Cell Physiol.

[pmed-0040234-b052] Szulgit G, Rudolph R, Wandel A, Tenenhaus M, Panos R (2002). Alterations in fibroblast alpha1beta1 integrin collagen receptor expression in keloids and hypertrophic scars. J Invest Dermatol.

[pmed-0040234-b053] Wu J, Ma B, Yi S, Wang Z, He W (2004). Gene expression of early hypertrophic scar tissue screened by means of cDNA microarrays. J Trauma.

[pmed-0040234-b054] Messadi DV, Le A, Berg S, Jewett A, Wen Z (1999). Expression of apoptosis-associated genes by human dermal scar fibroblasts. Wound Repair Regen.

[pmed-0040234-b055] Machesney M, Tidman N, Waseem A, Kirby L, Leigh I (1998). Activated keratinocytes in the epidermis of hypertrophic scars. Am J Pathol.

[pmed-0040234-b056] Moulin V, Larochelle S, Langlois C, Thibault I, Lopez-Valle CA (2004). Normal skin wound and hypertrophic scar myofibroblasts have differential responses to apoptotic inductors. J Cell Physiol.

[pmed-0040234-b057] Majno G, Gabbiani G, Hirschel BJ, Ryan GB, Statkov PR (1971). Contraction of granulation tissue in vitro: Similarity to smooth muscle. Science.

[pmed-0040234-b058] Ting SB, Caddy J, Hislop N, Wilanowski T, Auden A (2005). A homolog of Drosophila grainy
*head* is essential for epidermal integrity in mice. Science.

[pmed-0040234-b059] Frank DE, Carter WG (2004). Laminin 5 deposition regulates keratinocyte polarization and persistent migration. J Cell Sci.

[pmed-0040234-b060] Poole K, Khairy K, Friedrichs J, Franz C, Cisneros DA (2005). Molecular-scale topographic cues induce the orientation and directional movement of fibroblasts on two-dimensional collagen surfaces. J Mol Biol.

[pmed-0040234-b061] Zhang ZG, Bothe I, Hirche F, Zweers M, Gullberg D (2006). Interactions of primary fibroblasts and keratinocytes with extracellular matrix proteins: Contribution of alpha2beta1 integrin. J Cell Sci.

[pmed-0040234-b062] Firth JD, Putnins EE (2004). Keratinocyte growth factor 1 inhibits wound edge epithelial cell apoptosis in vitro. J Invest Dermatol.

[pmed-0040234-b063] Wilgus TA, Matthies AM, Radek KA, Dovi JV, Burns AL (2005). Novel function for vascular endothelial growth factor receptor-1 on epidermal keratinocytes. Am J Pathol.

[pmed-0040234-b064] Ridge KM, Linz L, Flitney FW, Kuczmarski ER, Chou YH (2005). Keratin 8 phosphorylation by protein kinase C delta regulates shear stress-mediated disassembly of keratin intermediate filaments in alveolar epithelial cells. J Biol Chem.

[pmed-0040234-b065] Tzima E, Irani-Tehrani M, Kiosses WB, Dejana E, Schultz DA (2005). A mechanosensory complex that mediates the endothelial cell response to fluid shear stress. Nature.

[pmed-0040234-b066] Nho RS, Xia H, Kahm J, Kleidon J, Diebold D (2005). Role of integrin-linked kinase in regulating phosphorylation of Akt and fibroblast survival in type I collagen matrices through a beta1 integrin viability signaling pathway. J Biol Chem.

[pmed-0040234-b067] Yano S, Komine M, Fujimoto M, Okochi H, Tamaki K (2004). Mechanical stretching in vitro regulates signal transduction pathways and cellular proliferation in human epidermal keratinocytes. J Invest Dermatol.

[pmed-0040234-b068] Grinnell F, Zhu M, Carlson MA, Abrams JM (1999). Release of mechanical tension triggers apoptosis of human fibroblasts in a model of regressing granulation tissue. Exp Cell Res.

[pmed-0040234-b069] Derderian CA, Bastidas N, Lerman OZ, Bhatt KA, Lin SE (2005). Mechanical strain alters gene expression in an in vitro model of hypertrophic scarring. Ann Plast Surg.

[pmed-0040234-b070] Xia H, Nho RS, Kahm J, Kleidon J, Henke CA (2004). Focal adhesion kinase is upstream of phosphatidylinositol 3-kinase/Akt in regulating fibroblast survival in response to contraction of type I collagen matrices via a beta 1 integrin viability signaling pathway. J Biol Chem.

[pmed-0040234-b071] Santoro MM, Gaudino G, Marchisio PC (2003). The MSP receptor regulates alpha6beta4 and alpha3beta1 integrins via 14-3-3 proteins in keratinocyte migration. Dev Cell.

[pmed-0040234-b072] du Roure O, Saez A, Buguin A, Austin RH, Chavrier P (2005). Force mapping in epithelial cell migration. Proc Natl Acad Sci U S A.

[pmed-0040234-b073] Galko MJ, Krasnow MA (2004). Cellular and genetic analysis of wound healing in Drosophila larvae. PLoS Biol.

[pmed-0040234-b074] Aarabi S, Bhatt KA, Shi Y, Paterno J, Chang EI (2007). Mechanical load initiates hypertrophic scar formation through decreased cellular apoptosis. FASEB J.

[pmed-0040234-b075] [No authors listed] (1978). On the anatomy and physiology of the skin. II. Skin tension by Professor K. Langer, presented at the meeting of 27th November 1861. Br J Plast Surg.

[pmed-0040234-b076] Scheid A, Wenger RH, Christina H, Camenisch I, Ferenc A (2000). Hypoxia-regulated gene expression in fetal wound regeneration and adult wound repair. Pediatr Surg Int.

[pmed-0040234-b077] Scheid A, Wenger RH, Schaffer L, Camenisch I, Distler O (2002). Physiologically low oxygen concentrations in fetal skin regulate hypoxia-inducible factor 1 and transforming growth factor-beta3. FASEB J.

[pmed-0040234-b078] Soo C, Beanes SR, Hu FY, Zhang X, Dang C (2003). Ontogenetic transition in fetal wound transforming growth factor-beta regulation correlates with collagen organization. Am J Pathol.

[pmed-0040234-b079] Takimoto E, Champion HC, Li M, Ren S, Rodriguez ER (2005). Oxidant stress from nitric oxide synthase-3 uncoupling stimulates cardiac pathologic remodeling from chronic pressure load. J Clin Invest.

[pmed-0040234-b080] Waghray M, Cui Z, Horowitz JC, Subramanian IM, Martinez FJ (2005). Hydrogen peroxide is a diffusible paracrine signal for the induction of epithelial cell death by activated myofibroblasts. FASEB J.

[pmed-0040234-b081] Renovo (2006). Results of clinical trials 1007/1011. http://www.renovo.co.uk/itemdetails.asp?s_id=11&news_id=25.

[pmed-0040234-b082] Van den Kerckhove E, Stappaerts K, Fieuws S, Laperre J, Massage P (2005). The assessment of erythema and thickness on burn related scars during pressure garment therapy as a preventive measure for hypertrophic scarring. Burns.

[pmed-0040234-b083] Gold MH (1994). A controlled clinical trial of topical silicone gel sheeting in the treatment of hypertrophic scars and keloids. J Am Acad Dermatol.

[pmed-0040234-b084] Xu XM, Sansores-Garcia L, Chen XM, Matijevic-Aleksic N, Du M (1999). Suppression of inducible cyclooxygenase 2 gene transcription by aspirin and sodium salicylate. Proc Natl Acad Sci U S A.

[pmed-0040234-b085] Li-Tsang CW, Lau JC, Choi J, Chan CC, Jianan L (2006). A prospective randomized clinical trial to investigate the effect of silicone gel sheeting (Cica-Care) on post-traumatic hypertrophic scar among the Chinese population. Burns.

[pmed-0040234-b086] Gold MH, Foster TD, Adair MA, Burlison K, Lewis T (2001). Prevention of hypertrophic scars and keloids by the prophylactic use of topical silicone gel sheets following a surgical procedure in an office setting. Dermatol Surg.

[pmed-0040234-b087] Jih MH, Friedman PM, Kimyai-Asadi A, Goldberg LH (2004). Successful treatment of a chronic atrophic dog-bite scar with the 1450-nm diode laser. Dermatol Surg.

[pmed-0040234-b088] Alster TS (1999). Clinical and histologic evaluation of six erbium: YAG lasers for cutaneous resurfacing. Lasers Surg Med.

[pmed-0040234-b089] Tse Y, Ostad A, Lee HS, Levine VJ, Koenig K (1996). A clinical and histologic evaluation of two medium-depth peels. Glycolic acid versus Jessner's trichloroacetic acid. Dermatol Surg.

[pmed-0040234-b090] Chen JS, Shack RB, Reinisch L, Spector N, Zinsser JW (2001). A comparison of scar revision with the free electron and carbon dioxide resurfacing lasers. Plast Reconstr Surg.

[pmed-0040234-b091] Silverstein P (1997). Depressed scar revision. LifeCell Clinical Case Summary.

[pmed-0040234-b092] Integra LifeSciences (2007). Results of Multicenter Safety and Efficacy Clinical Trial (Pivotal Study). http://www.integra-ls.com/products/?product=46.

[pmed-0040234-b093] Carsin H, Ainaud P, Le Bever H, Rives J, Lakhel A (2000). Cultured epithelial autografts in extensive burn coverage of severely traumatized patients: A five year single-center experience with 30 patients. Burns.

[pmed-0040234-b094] Compton CC (1992). Current concepts in pediatric burn care: The biology of cultured epithelial autografts: An eight-year study in pediatric burn patients. Eur J Pediatr Surg.

